# Questions on Medicines, Alcohol, and Illicit Drug Interactions in the Finnish AddictionLink Online Service: A Descriptive Analysis

**DOI:** 10.1177/14550725261463002

**Published:** 2026-07-06

**Authors:** Mari Vaarankorpi, Ville Valkonen, Niina Karttunen

**Affiliations:** 1 School of Pharmacy, 205537 University of Eastern Finland, Kuopio, Finland; 2 Wellbeing Services County of North Savo, Hospital Pharmacy, Kuopio, Finland

**Keywords:** medicines, drugs, illicit substances, alcohol, drug interactions, questions

## Abstract

**Aims:**

To explore which drugs and intoxicants people most commonly seek information about from the Finnish AddictionLink online information service and to describe the types of the drug interaction questions submitted, the most frequently mentioned substances in the questions and the background characteristics of the questioners.

**Methods:**

The study was conducted using data from the Finnish AddictionLink online information service. Medication-related questions submitted between 2011 and 2023 (*n* = 1858) were extracted. Interaction-related questions (*n* = 242) and the substances mentioned were categorized and analyzed. Background characteristics were described for individuals who submitted drug interaction-related questions (*n* = 197) and for those who submitted other medication-related questions (*n* = 1070).

**Results:**

Most questions concerned interactions between medications and intoxicants, with alcohol being the most frequently mentioned intoxicant, followed by stimulants and cannabis. Antidepressants were the most frequently mentioned drug class, followed by opioids, cardiovascular drugs, and benzodiazepines. Participants aged 13–25 years and those aged 46 years or older appeared to submit interaction-related questions more frequently, whereas individuals aged 26–45 were more likely to submit other medication-related questions.

**Conclusions:**

Questions concerning the concurrent use of medications with alcohol or illicit substances highlight a clear need for accessible and understandable information on drug–intoxicant interactions. The frequent involvement of antidepressants and other commonly used medications underscores the relevance of these interactions in routine treatment. Increasing awareness among both healthcare professionals and medication users is essential to support safer treatment choices.

## Introduction

Drugs and intoxicants have several clinically significant interactions that may alter the therapeutic efficacy of medications or cause severe adverse effects (Abbott et al., 2020; Lindsey et al., 2012). These interactions occur when one substance alters the pharmacodynamics or pharmacokinetics of another during concurrent use (Sun et al., 2023). Pharmacodynamic interactions involve changes in pharmacological effects, whereas pharmacokinetic interactions affect absorption, distribution, metabolism, and excretion (Niu et al., 2019; Palleria et al., 2013). Pharmacokinetic interactions cause the majority of adverse interactions (Abbott et al., 2020). Importantly, these interactions are bidirectional because drugs can influence intoxicants and vice versa.

Individuals may intentionally combine substances to cope with mental or physical health problems or to modify their effects; for example, to enhance the desired effects, to experience new effects or to reduce undesired effects (Boileau-Falardeau et al., 2022; van Amsterdam et al., 2023). Different combinations of substances might also be used according to availability and in illicit markets substances might be contaminated with other substances without the purchaser knowing. In addition, intoxicants are often used by individuals who are concurrently taking drugs prescribed by physicians for various medical conditions. Using multiple substances simultaneously (i.e., polysubstance use) increases the risk of acute toxicity and is often involved in fatal overdoses (Boileau-Falardeau et al., 2022; Figgatt et al., 2021). It is also associated with psychiatric and medical complications, poorer mental health, medical and substance use outcomes, and deficits of cognitive functioning (Timko et al., 2018).

Alcohol is the most widely used intoxicant globally, with 2,5 billion people aged at least 15 years reporting alcohol use in 2019 (Global Status Report on Alcohol and Health and Treatment of Substance Use Disorders, 2024). The use of illicit substances is less prevalent, but it has been increasing over the past decade. It is estimated that 292 million people aged 15–64 years used psychoactive drugs in 2022 (United Nations Office on Drugs and Crime, 2024). Attitudes and regulatory approaches towards illicit substances have also become less strict in many regions. Cannabis is the most commonly used illicit substance (although not considered illicit in all countries), with an estimated 228 million people who used it in the past year, followed by opioids (60 million), amphetamine-type stimulants (30 million) and cocaine (23 million). In addition, non-medical use of prescription drugs such as benzodiazepines and gabapentinoids has been documented across Europe and is considered an emerging concern, particularly in light of recent reports describing misuse and related harms (Hockenhull et al., 2021). Polysubstance use is common among those who use psychoactive substances, and the patterns of use are becoming more dynamic and complex, further elevating interaction risks (European Union Drugs Agency, 2024; United Nations Office on Drugs and Crime, 2024). In Finland, patterns of illicit substance use differ from many other countries, particularly regarding opioids. Buprenorphine has dominated the Finnish illicit opioid market for decades, and heroin use has remained comparatively rare (European Union Drugs Agency, 2024; Hakkarainen & Kainulainen, 2021). Similarly, benzodiazepines, especially alprazolam, and pregabalin are commonly found on the Finnish illicit market (European Union Drugs Agency, 2024).

Despite the clinical relevance of drug–intoxicant interactions, available information remains limited. Existing studies often focus on a narrow set of combinations or rely on a limited number of case reports (Abbott et al., 2020). Furthermore, the information that is freely accessible to the public varies considerably. Digital health services play an important role in providing low threshold support for individuals concerned about substance use. Among these services, AddictionLink (https://paihdelinkki.fi/en) is a Finnish online platform that has provided comprehensive, publicly accessible information on alcohol, drugs and behavioral addictions since 1996 and serves as a source of guidance for individuals seeking reliable substance related information. The website is maintained by the A Clinic Foundation and includes an online service where individuals can submit anonymous questions that are answered by professionals, including nurses, pharmacists, social workers or a psychologist. If requested by the questioner, the response can also be provided by a trained expert with relevant experiential knowledge. Although various types of digital resources, such as telephone-based helplines and general informational platforms, are available internationally, written asynchronous question and answer services comparable to AddictionLink information service are not commonly offered.

To better understand the demand for information on interactions, it is important to examine the questions submitted to the service. The purpose of this study was to explore which drugs and intoxicants people most commonly seek information about in the AddictionLink online service regarding potential interactions. In addition, the study aimed to describe the types of interaction-related questions, determine the most commonly mentioned substances, and describe the background characteristics of individuals who submitted the questions.

## Methods

In the AddictionLink service, all questions are saved in full text and categorized according to the content in a searchable database. Medication-related questions form one thematic category alongside others such as gaming and gambling problems, mental health issues, sexuality, family relationships, and substance use related concerns. The service receives approximately 700–1,000 questions annually across all categories, with medication questions constituting part of these submissions and interaction-related queries representing a smaller subset within that group. Personal details are not collected, but the questioner can voluntarily fill in general background details such as age, sex, education level, occupation, and place of residence. Some submissions include follow up questions.

**Figure 1.**
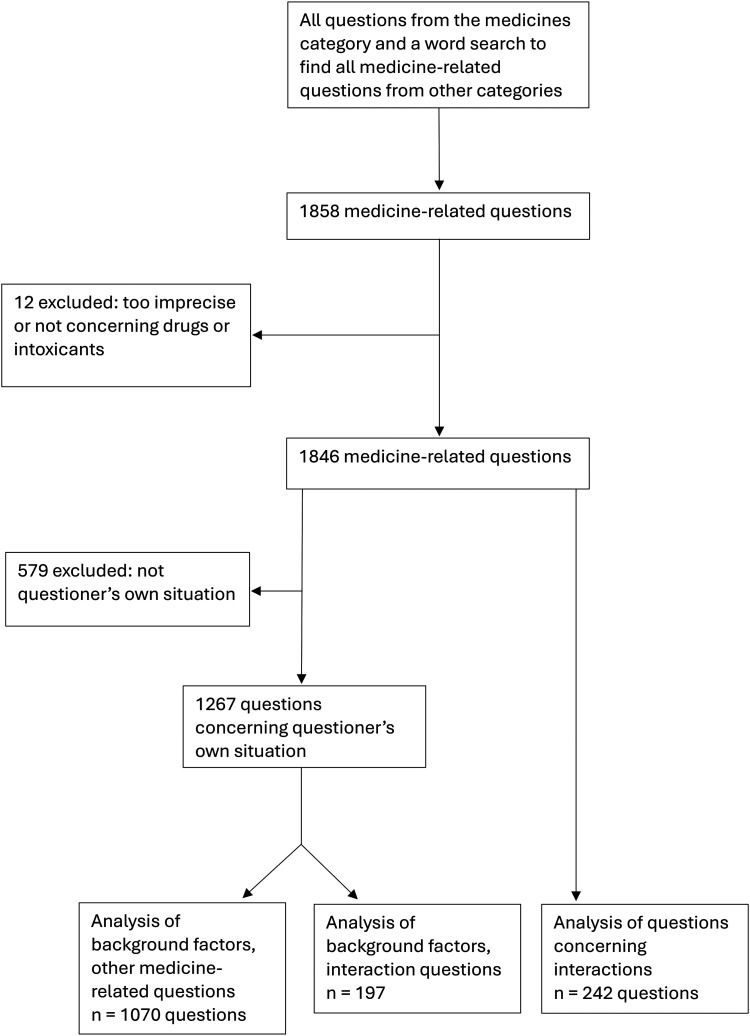
Data extraction process from the AddictionLink online information service database for drug interaction questions submitted between 2011 and 2023.

All questions from the “medicines” category were extracted, and a keyword search using terms such as “medicine” and “polysubstance use” was applied to identify medicine-related questions from other categories. Additional searches using common synonyms, drug classes, and brand names did not yield new questions. During the study period from 2011 to 2023, a total of 1858 medicine-related questions were submitted. Twelve questions were excluded due to imprecise content or because the question concerned chemicals unrelated to drugs or intoxicants (e.g., hair spray or hand sanitizer) (Figure 1). Of the remaining questions, 242 addressed interactions, and were analyzed in detail. Most interaction-related questions followed a relatively uniform structure, typically asking whether certain substances interact and whether the combination is risky. These interaction-related questions were classified into four categories according to the type of drug interaction, using the same group headings as Abbott et al. (2020): interactions between drugs; between drugs and alcohol; between drugs and illicit substances; and between drugs, alcohol, and illicit substances. These headings are used throughout the article to refer to the categories. To minimize errors in the background data, only questions referring to the questioner's own situation (*n* = 1267) were included in the descriptive table of background characteristics, whereas questions concerning other situations were excluded.

### Sociodemographic Characteristics of the Questioners

The variables describing the sender of the question were sex (male, female, other), age (years) (13–25, 26–35, 36–45, 46–55, 56+), education level (basic, including ≤10 years; secondary, including high school and vocational school; and higher, including all education beyond secondary), occupation (unemployed/stay at home parent, employed, pensioner, student) and place of residence (Helsinki metropolitan area, other areas). The question form of the AddictionLink information service was updated during the study period, which limited the use of the residence variable and prevented a more detailed regional classification. For the background characteristics, the number of observations (*n*) varies across variables due to the real-world nature of the data. Missing values were not imputed, and therefore no statistical analyses were conducted for these variables. Descriptive results are presented using available data only. The variables describing the content of the question were the type of interaction; the drugs and drug classes mentioned; whether alcohol or illicit substances were mentioned; and when applicable, the specific illicit substances and their classes. The mentioned substances were categorized according to the categories presented in Table 1, and rarely mentioned drugs were categorized as “other”. Several substances commonly misused in Finland, such as buprenorphine, benzodiazepines and pregabalin, can be obtained both legally by prescription and illegally on the illicit market. Because the dataset contained only the names of substances mentioned in submitted questions and did not include information on the source of the substance or the questioner's purpose (medical vs. non-medical use), it was not possible to distinguish between prescription use and illicit use. For this reason, these substances were categorized as drugs. In this dataset, smoking was mentioned only in a few questions, mainly in relation to smoking cessation medications, and was therefore not examined separately as an independent substance, even though it may have interactions with some substances via cytochrome P450 (CYP450)-mediated metabolism.

**Table 1.** Content of Substance Categories in the Variables Describing the Questions.

Category	Included Anatomical Therapeutic Chemical (ATC) Classification groups and substances
*Drugs*	
ADHD drugs	N06BA
Alcohol/nicotine withdrawal drugs	N07BA, N07BB
Antidepressants	N06A
Antiepileptics	N02BF, N03AF, N03AG, N03AX
Antimicrobials	J01–J05
Antipsychotics	N05A
Benzodiazepines and related drugs	N03AE, N05BA, N05CD, N05CF
Cardiovascular drugs	C01-10, B01A
Non-opioid analgesics	N02BA, N02BE, M01A
Opioids	N02A, N07BC
*Illicit substances*	
Stimulants	Cocaine, amphetamine, methamphetamine, MDMA
Cannabis	Cannabis
Hallucinogens	Psilocybin, LSD
Heroin	Heroin

### Statistical Analysis

The data were analyzed with SPSS, version 29 (IBM Corp.) and the graphics (bar and pie charts) were created with Excel, version 16.89 (Microsoft Corp.). Background characteristics of the questioners were summarized in a descriptive table showing frequencies and percentages according to whether the question concerned interaction questions or other drug-related questions. Crosstabulation with Pearson's chi-square test was used to analyze associations between the type of interaction questions (drug–alcohol, drug–illicit drug, drug–drug) and the most frequently mentioned drug classes in the questions. *p* < .05 was considered statistically significant.

### Research Ethics

A research permit from the A-Clinic Foundation was required for the study. The permit was applied for in November 2023 and granted in February 2024. All researchers signed a nondisclosure agreement provided by the A-Clinic Foundation prior to the data analysis. The nondisclosure agreement requires the researchers to comply with the Foundation's data processing and storage practices. According to the Finnish National Ethics Committee, ethical review was not required because the material was fully anonymous (TENK, 2019). The questioners accepted the terms and conditions of the AddictionLink information service when submitting their questions. The terms and conditions include permission to use the submitted questions and background information for research purposes.

## Results

**Figure 2.**
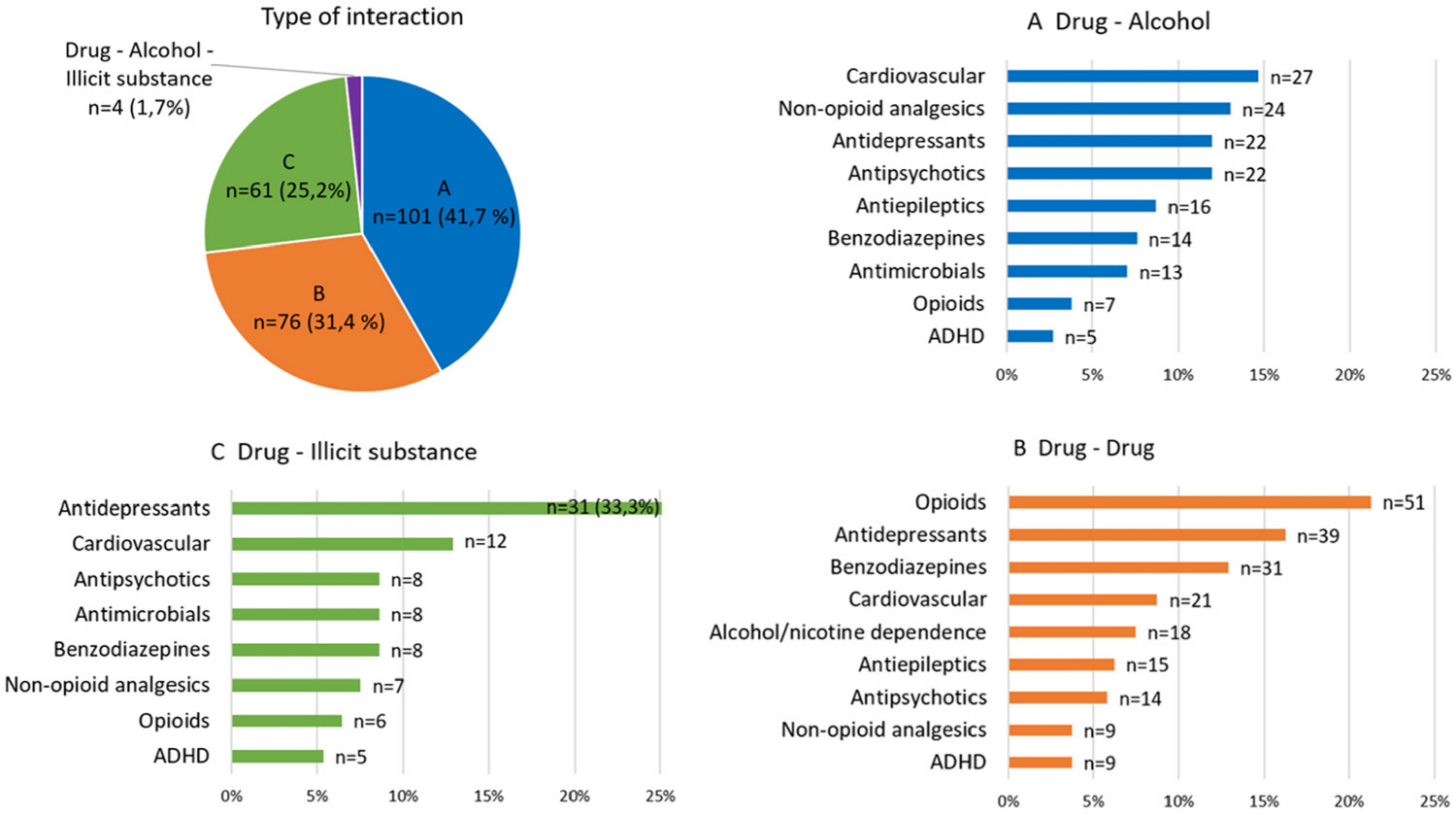
Interaction-related questions (n=242) submitted to the AddictionLink online information service (2011–2023) categorized by interaction type. Drugs mentioned in these questions (n=517) are further classified by interaction type and drug class.

The majority of interaction-related questions concerned drugs and intoxicants (68.6%), including alcohol (41.7%), illicit substances (25.2%), or both (1.7%) (Figure 2). Slightly less than one-third concerned drug interactions between two or more drugs (31.4%). Among the 65 questions regarding interactions with illicit substances, stimulants were mentioned most frequently (53.8%, *n* = 35), followed by cannabis (33.8%, *n* = 22) and hallucinogens (10.8%, *n* = 7). Only one question (1.5%) concerned heroin. Over the study period, the proportion of questions related to drugs and intoxicants increased from approximately 2% to 13% of all interaction-related questions.

Because several drugs could be mentioned in a single question, the total number of drugs mentioned (*n* = 517) exceeded the total number of questions (*n* = 242) (Figure 2 and Table 2). Cardiovascular drugs (14.7%), non-opioid analgesics (13.0%), antidepressants (12.0%), and antipsychotics (12.0%) were the most often mentioned drug classes in drug-alcohol interaction questions. Non-opioid analgesics and antiepileptics were mentioned significantly more often in drug–alcohol interaction questions than in other interaction types.

**Table 2.** Associations Between Interaction Question Types and the Most Commonly Mentioned Drug Classes in Questions Submitted to the Finnish AddictionLink Information Service (2011–2023).

	Drug–Drug (*n* = 240), *n* (%)	Drug–Alcohol (*n* = 184), *n* (%)	Drug–Illicit Substance (*n* = 93), *n* (%)	Total (*n* = 517), *n* (%)^a^	*p* ^b^
Antidepressants	39 (16.2)	22 (12.0)	31 (33.3)	92 (17.8)	<.001
Opioids	51 (21.2)	7 (3.8)	6 (6.5)	64 (12.4)	<.001
Cardiovascular	21 (8.8)	27 (14.7)	12 (12.9)	60 (11.6)	0.701
Benzodiazepines and related drugs	31 (12.9)	14 (7.6)	8 (8.6)	53 (10.3)	<.001
Antipsychotics	14 (5.8)	22 (12.0)	8 (8.6)	44 (8.5)	.397
Non-opioid analgesics	9 (3.8)	24 (13.0)	7 (7.5)	40 (7.7)	.031
Antiepileptics	15 (6.3)	16 (8.7)	2 (2.2)	33 (6.4)	.041
Alcohol/nicotine withdrawal	18 (7.5)	3 (1.6)	3 (3.2)	24 (4.6)	<.001
Antimicrobials	2 (0.8)	13 (7.1)	8 (8.6)	23 (4.4)	.020
ADHD	9 (3.8)	5 (2.7)	5 (5.4)	19 (3.7)	.378
Other^c^	31 (12.9)	31 (16.8)	3 (3.2)	65 (12.6)	NA

a Many questions mentioned multiple drugs, and so the total number of drugs exceeded the number of questions.

b *p* < .05 indicates statistically significant differences in the distribution of each drug class across interaction types, based on Pearson's chi-squared test.

c Drug classes with very few mentions were excluded from the *p*-value analysis.

NA, not available.

Antidepressants (33.3%) were clearly the most frequently mentioned drug class in questions concerning drug-illicit substances interactions, followed by cardiovascular drugs (12.9%) (Figure 2 and Table 2). In addition, antidepressants were mentioned significantly more often in drug–illicit substance questions compared to other interaction types. Stimulants were mentioned in almost half (45.2%) of the questions concerning interaction between antidepressants and illicit substances. Antiepileptics were significantly less frequent in drug-illicit substance interaction questions than in other categories.

Opioids (21.2%), antidepressants (16.2%), and benzodiazepines (12.9%) were the most often mentioned drug classes in questions regarding interactions between drugs (Figure 2 and Table 2). Opioids, benzodiazepines and drugs for alcohol or nicotine withdrawal were mentioned relatively more often in drug–drug interaction questions than in other interaction types. In contrast, antimicrobials were significantly less frequently mentioned in drug–drug interaction questions compared to other categories.

Additional details on the substances mentioned across the different interaction categories are provided in the Supplementary material (Tables S1 and S2), illustrating the wide range of medications and intoxicants involved in the questions and highlighting the diversity of drug combinations for which users sought information.

The background characteristics of the questioners were largely similar when comparing those who submitted drug interaction questions with those who submitted other drug-related questions (Table 3). The largest differences were observed in the age of the questioners. Teenagers and young adults aged 13–25 years (26.4%) appeared to submit interaction questions more frequently than other age groups. Individuals aged 26–45 years (54%) more commonly submitted other drug-related questions, whereas those aged 46 years and older more often submitted questions concerning drug interactions. The difference by sex was very small, but females submitted slightly more interaction questions than males. Because background information was voluntary, missing values were common, and repeated submissions by the same individual could not be excluded, these findings should be interpreted descriptively.

**Table 3.** Descriptive Characteristics of Questioners Who Submitted Drug Interaction Questions and Other Drug-Related Questions to the Finnish AddictionLink Online Information Service (2011–2023).

	Interaction Questions (*n* = 197), *n* (%)	Other Drug-Related Questions (*n* = 1070), *n* (%)
*Sex*	176	998
Male	75 (42.6)	456 (45.7)
Female	101 (57.4)	542 (54.3)
*Age (years)*	178	1013
13–25	47 (26.4)	201 (19.8)
26–35	36 (20.2)	286 (28.2)
36–45	26 (14.6)	261 (25.8)
46–55	36 (20.2)	148 (14.6)
56+	33 (18.6)	117 (11.6)
*Education*	156	947
Basic	23 (14.7)	208 (22.0)
Secondary	83 (53.2)	440 (46.4)
Higher	50 (32.1)	299 (31.6)
*Occupation*	113	803
Unemployed/stay at home parent	27 (23.9)	240 (29.9)
Employed	37 (32.7)	274 (34.1)
Pensioner	28 (24.8)	154 (19.2)
Student	21 (18.6)	135 (16.8)
*Residence*	158	887
Helsinki metropolitan area	37 (23.4)	224 (25.3)
Other areas	121 (76.6)	663 (74.7)

The number of observations varies between variables because background information was voluntary and missing values were not imputed. The data are descriptive, and no statistical comparisons were performed.

## Discussion

In this study, we examined questions submitted to the AddictionLink online information service and found that the majority of interaction-related questions concerned drug-intoxicant interactions, followed by interactions between different drugs. The most frequently mentioned drug groups overall were antidepressants, opioids, cardiovascular drugs and benzodiazepines. A closer examination of the drugs mentioned showed that questions most often involved widely used or clinically significant drug classes, particularly those associated with non-medical use or known for their interaction potential. Questions about interactions involving illicit substances most commonly concerned stimulants or cannabis. In interpreting these findings, it is important to consider the Finnish context. A plausible explanation for why the questions more often concerned interactions between legally available medications rather than illicit substances is that several substances commonly used recreationally in Finland are prescription medicines. Consequently, some questions categorized as drug–drug interactions may in practice relate to the non-medical or intoxicating use of prescription medications.

Our findings indicate that individuals who use psychoactive substances do have situations in which accessible and reliable information on the risks of combining different substances is needed. Such information is not readily available through existing public sources, which often offer only limited or fragmented guidance. In the absence of credible and comprehensible information, people may turn to informal channels, including peers, social media, and open online forums, where the advice shared is frequently incomplete, anecdotal or inaccurate. This highlights the need for a service that provides easily accessible, evidence-based, and easily understandable information on substance interaction risks.

### The Most Common Drug Classes in Interaction Questions

Antidepressants were the most frequently mentioned drug class overall, despite the AddictionLink website focusing primarily on intoxicants. With the exception of bupropion and venlafaxine, antidepressants are rarely used as intoxicants (Schifano et al., 2018). The large number of questions concerning interactions between intoxicants and antidepressants might refer to questioners’ mental health issues because these conditions are commonly treated with antidepressants. Substance use disorders often co-occurs with other psychiatric conditions, including mood, stress-related, neurotic, and somatoform disorders (Currie et al., 2005; Evans & Sullivan, 2014; Plana-Ripoll et al., 2020; Volkow & Blanco, 2023). The relationship between substance use and mental health is considered bidirectional (i.e., substance use may contribute to the development of mental health problems), whereas psychiatric disorders can increase the risk of substance use (Hunt et al., 2020; Srifuengfung et al., 2024). Due to these overlaps, the number of questions related to antidepressants is not surprising, and it is encouraging that people seek information about the safety of the medications and substances they use. However, this also raises concerns because antidepressants can have potentially dangerous interactions with other drugs and substances, such as benzodiazepines, opioids or stimulants (Leonard et al., 2021; Lindsey et al., 2012; Pilgrim et al., 2010; Sarparast et al., 2022; Thomas et al., 2015). These interactions may lead to sedation, serotonin toxicity or even death.

Opioids are widely used for both medical and non-medical purposes and were the second most frequently mentioned drug class, with most questions concerning drug–drug interactions and fewer involving alcohol or illicit substances. This finding aligns with previous literature, as opioids are often co-used with other substances, although the distribution observed here may differ due to classification criteria (Degenhardt et al., 2019; Figgatt et al., 2021; van Amsterdam et al., 2023). Opioids have a narrow therapeutic index, significant interindividual variability, and can cause severe toxicity, making them highly susceptible to adverse effects when combined with other substances (Aapro et al., 2024; Coates & Lazarus, 2023; Overholser & Foster, 2011). Using other substances simultaneously with opioids greatly increases the risks of opioid overdose (Degenhardt et al., 2019).

Many commonly used opioids, such as oxycodone, tramadol, hydrocodone, fentanyl, codeine, and methadone, are metabolized by CYP450 enzymes, which makes them prone to pharmacokinetic interactions with substances affecting CYP450 activity (Coates & Lazarus, 2023; Overholser & Foster, 2011). Opioid effects may be either potentiated or diminished by such interactions. Additionally, opioids are central nervous system depressants, and their concurrent use with other central nervous system (CNS) depressants (e.g., benzodiazepines, muscle relaxants, and antipsychotics) can lead to pharmacodynamic interactions causing respiratory depression and even death (Coates & Lazarus, 2023). Despite these risks, alcohol, benzodiazepines, and gabapentinoids are frequently co-used with opioids and commonly implicated in overdose deaths (Bachhuber et al., 2016; Degenhardt et al., 2019; Hwang et al., 2016; Powell et al., 2023). As a result of to its serotonergic properties, tramadol may interact with other serotonergic drugs, such as some antidepressants, in a manner that increases the risk of serotonergic adverse effects. This concern is considered specific to tramadol, as opioids lacking serotonergic activity are not generally associated with comparable interaction risks (Park et al., 2014; Pilgrim et al., 2010).

Benzodiazepines and related drugs, which have been widely used sedative and anxiolytic drugs for long, were the fourth most mentioned drug class over all in this study (Hockenhull et al., 2021). Non-medical use of benzodiazepines and related drugs is common, particularly among individuals with substance use disorders, those who misuse other substances, and those with psychiatric symptoms (McHugh et al., 2020; Russell et al., 2023; Votaw et al., 2019). In addition, “designer” benzodiazepines and illicit substances adulterated with benzodiazepines are present on the illicit market. Over one-quarter of benzodiazepine-related questions concerned interactions with alcohol, highlighting a significant overdose risk associated with concurrent use (Kleinman & Weiss, 2022). Benzodiazepines have additive sedative effects when combined with drug classes frequently mentioned in the questions such as opioids, antipsychotics, and certain antidepressants (Bénard-Laribière et al., 2016). Moreover, many benzodiazepines are metabolized by CYP3A4 and CYP2C19 enzymes, making them susceptible to pharmacokinetic interactions with enzyme inhibitors such as azole antifungals, certain calcium channel blockers, and selective serotonin reuptake inhibitors, which may potentiate benzodiazepine effects (Dubovsky & Marshall, 2022).

In Finland, several opioids and benzodiazepines are closely linked to non-medical use and their availability on the illicit market. In this context, it is understandable that many interaction related questions focused on drug–drug interactions. The prominent role of these prescription medications in Finnish non-medical use likely contributes to the observation that some questions classified as drug–drug interactions reflect concerns arising from the non-medical use of medications, rather than intentional combinations of legally prescribed drugs taken as directed.

### Drug Interaction Questions Related to Intoxicants

Alcohol is the most used intoxicant and majority of the questions were concerning interactions between alcohol and drugs. Cardiovascular drugs were the most frequently mentioned drug class in questions regarding interactions with alcohol, and this may be because they include a wide variety of drugs, and their use is overall very common. Although these drugs are not usually combined with alcohol for non-medical purposes, concurrent use poses risks. In older adults, heavy alcohol consumption with cardiovascular medication may increase the risk of orthostatic hypotension and major bleeding (Holton et al., 2017). In addition to cardiovascular drugs, certain antimicrobials and non-opioid analgesics were often mentioned in alcohol interaction questions, possibly because these drug classes are well known for their potential interactions with alcohol (Chan & Anderson, 2014). Chronic alcohol use increases the risk of paracetamol-induced hepatotoxicity (Chidiac et al., 2023) and a disulfiram-like reaction has been associated with concurrent use of metronidazole and alcohol, although evidence remains inconclusive (Feldman & Jaszczenski, 2023; Visapää et al., 2002). Alcohol is a CNS depressant, and co-use with other CNS depressants, such as antipsychotics, antiepileptics, and benzodiazepines, may result in severe interactions, including fatal outcomes (Gudin et al., 2013; Thomas et al., 2021).

Most often mentioned illicit substances were stimulants followed by cannabis, even though cannabis is more commonly used. A third of the questions concerning drug interactions with illicit substances mentioned antidepressants as the drug, almost half of which were concerning interactions between antidepressants and stimulants. Simultaneous use of antidepressants and stimulants may cause interactions such as serotonin toxicity, hypertensive crisis or even death (Pilgrim et al., 2010). The regulatory status of cannabis varies around the world, and it is used both for medical and non-medical purposes (Nachnani et al., 2024). Available information of drug interactions with cannabis is very limited and centred around the drugs used for similar indications as medicinal cannabis. The diversity of marijuana strains and products in addition to several different routes of administration makes studying drug interactions with cannabis challenging (Cox et al., 2019). Cannabinoids are metabolized by CYP450 enzymes and may also inhibit certain CYP450 enzymes, possibly leading to interactions with medications (Gilmartin et al., 2021; Ho et al., 2024; Lopera et al., 2022). Pharmacokinetic drug interactions with cannabis may be problematic especially with low therapeutic level drugs. Case reports suggest that cannabis use with warfarin might lead to increased international normalized ratio values due to inhibition of warfarin metabolism (Damkier et al., 2019; Hsu & Painter, 2020). Cannabis co-use has also been associated with elevated concentrations of several drugs, including opioids, antiepileptics, and immunosuppressants used to prevent transplant rejection (Ben-Menachem et al., 2020; Ebrahimi-Fakhari et al., 2020; Gaston et al., 2017; Geffrey et al., 2015; Leino et al., 2019; Vierke et al., 2021). Increased drug concentrations may result in adverse effects such as diarrhea, drowsiness, and sedation.

### Sociodemographic Characteristics of the Questioners

Over one-fourth of the interaction-related questions were submitted by individuals aged 13–25 years. This age group is particularly vulnerable to experimenting with substances, and early initiation of substance use is associated with polysubstance use (Aly et al., 2020; Gao et al., 2023). However, because background information was voluntary and repeated submissions by the same individual could not be excluded, this observation should be interpreted cautiously. In contrast, individuals aged 56 years and older submitted more interaction-related questions compared to other drug-related questions. This may reflect the higher prevalence of polypharmacy among older adults, which increases the relevance of drug interaction concerns (Barghouth et al., 2023).

Females submitted slightly more interaction-related questions than males, and it is noteworthy because the use of intoxicants is more common in males (United Nations Office on Drugs and Crime, 2024). However, women often experience faster progression of substance use and face disproportionate health consequences, social stigma, and barriers to treatment access. For example, these barriers include logistical challenges such as lack of childcare and stigmatization, particularly for pregnant women and mothers (Apsley et al., 2023). Because AddictionLink is an anonymous online service, these barriers do not apply, potentially making the platform more accessible for women. Anonymity may also lower the threshold for other groups who face stigma or social risks when seeking help. Recent research on relational anonymity shows that the need for anonymity is often linked to societal stigma, exclusionary practices or fears of negative consequences from recording illicit drug use (Ranta et al., 2024). Anonymity is therefore a critical response to systemic challenges, ensuring that individuals can seek help without fear of sanctions or discrimination.

Although several freely accessible drug interaction tools exist, including databases that allow the checking of interactions between medications and alcohol, these resources are primarily designed for medication focused queries and offer limited coverage of interactions involving psychoactive substances used for intoxicating purposes. Professional drug interaction databases used in healthcare settings provide more comprehensive and clinically validated information, but comparable high-quality resources are not available to lay users. In addition, most openly available interaction tools are provided in English, which may further limit their accessibility. This context highlights the need for publicly accessible, user friendly, and context specific guidance alongside services such as AddictionLink.

### Strengths and Limitations

The study has several strengths, but also some limitations. The AddictionLink website is an easily accessible online service that provides free and independent information on intoxicants and addiction nationwide. Internet is widely used, and the question form is easy to use, allowing the service reach a broad and diverse group of people. The service is anonymous, making it approachable to all individuals, including those who are unwilling to reveal their identity when seeking help for substance use. Data are collected from free-form communication submitted by the questioners, providing detailed information about the individual's situation from their own perspective. However, because the service was designed for support and counselling rather than for research purposes, it does not fully meet the needs of scientific research. The form for collecting background information is simple and completing it is voluntary. Incorrectly completed or missing background information may affect the generalizability of our results. When questioners ask about another person's situation, they may provide that person's background information instead of their own. For this reason, questions concerning other person's situation were excluded from the descriptive table of background characteristics. Follow-up questions were not included in the analysis of background variables, but it is possible that some individuals submitted later questions as new questions, in which case their information may appear more than once in the background data. Due to the nature of the data, the descriptive summaries may not fully capture the characteristics of all service users, and no firm conclusions can be drawn. The values of some variables may be underrepresented because the questioners decide what information they share, which means that some relevant information may not be included. When interpreting the results, it is important to consider that many drugs, such as opioids, benzodiazepines, gabapentinoids, and ADHD (i.e. attention deficit hyperactivity disorder) medications, are used for both medical and non-medical purposes. Based on the data, it is not possible to distinguish whether the use is medical or non-medical, due to which they have been classified as drugs. The service is not accessible to individuals without internet access. Finally, as the questions are submitted in free form, the interpretation of the questions may affect how they are classified.

## Conclusions

The majority of drug interaction-related questions submitted to the AddictionLink online information service concerned the concurrent use of medications with alcohol or illicit substances. Antidepressants, opioids, cardiovascular drugs, and benzodiazepines were among the most frequently mentioned drug classes, whereas stimulants and cannabis were the most common illicit substances in interaction-related questions. These findings indicate a need for accessible, evidence-based, and understandable information on interactions between medications and intoxicants. Improving awareness among both healthcare professionals and people who use medications may help reduce harmful substance combinations and support safer treatment choices. Future research should focus on identifying gaps in available interaction information and on how well such knowledge is accessed and applied by both healthcare professionals and people using medications.

## Supplemental Material

sj-docx-1-nad-10.1177_14550725261463002 - Supplemental material for Questions on Medicines, Alcohol, and Illicit Drug Interactions in the Finnish AddictionLink Online Service: A Descriptive Analysis Supplemental material, sj-docx-1-nad-10.1177_14550725261463002 for Questions on Medicines, Alcohol, and Illicit Drug Interactions in the Finnish AddictionLink Online Service: A Descriptive Analysis by Mari Vaarankorpi, Ville Valkonen and Niina Karttunen in Nordic Studies on Alcohol and Drugs
