# Supplementary material for: Questions on Medicines, Alcohol, and Illicit Drug Interactions in the Finnish AddictionLink Online Service: A Descriptive Analysis
Source: Nordisk Alkohol Nark. 2026 Jul 6:14550725261463002. Online ahead of print. doi: 10.1177/14550725261463002 (PMC13337570; doi:10.1177/14550725261463002)
Supplement: sj-docx-1-nad-10.1177_14550725261463002 - Supplemental material for Questions on Medicines, Alcohol, and Illicit Drug Interactions in the Finnish AddictionLink Online Service: A Descriptive Analysis [file sj-docx-1-nad-10.1177_14550725261463002.docx]

**Supplemental material** for **“**Questions on medicines, alcohol, and illicit drug interactions in the Finnish AddictionLink online service: a descriptive analysis” by Vaarankorpi M, Valkonen V, and Karttunen N in Nordic Studies on Alcohol and Drugs.

**Supplementary table 1**. Most frequently mentioned CNS (central nervous system) and other drugs in drug-intoxicant interaction questions.

| **CNS drugs** | **Alcohol** | **Cannabis** | **Stimulants** | **Hallucinogens** |
| --- | --- | --- | --- | --- |
| **ADHD drugs** n=10 | Lisdexamphetamine (2) Atomoxetine Dexamphetamine Methylphenidate | Methylphenidate (2) | Methylphenidate (2) | Methylphenidate |
| **Antidepressants** n=53 | Vortioxetine (4) Mirtazapine (4) Es/citalopram (3) Duloxetine (2) Sertraline (2) Amitriptyline Bupropion Buspirone Doxepin Fluoxetine Moclobemide Paroxetine | Duloxetine (2) Es/citalopram (2) Amitriptyline Mirtazapine Sertraline Venlafaxine Vortioxetine | Venlafaxine (4) Vortioxetine (4) Bupropion (3) Es/citalopram (2) Doxepin Moclobemide Sertraline | Es/citalopram (2) Vortioxetine (2) Sertraline Venlafaxine |
| **Antiepileptics** n=18 | Gabapentin (6) Pregabalin (4) Sodium valproate (3) Lamotrigine (2) Topiramate |  | Lamotrigine (2) |  |
| **Antipsychotics** n=30 | Quetiapine (10) Flupentixol (2) Levomepromazine (2) Lithium (2) Olanzapine (2) Ziprasidone (2) Aripiprazole Risperidone | Clozapine Haloperidol Quetiapine | Quetiapine (3) Lithium Risperidone |  |
| **Benzodiazepines**  **and related drugs** n=22 | Oxazepam (5) Temazepam (4) Chlordiazepoxide Clonazepam Diazepam Lorazepam Zolpidem | Alprazolam Lorazepam | Diazepam (2) Alprazolam Oxazepam Temazepam | Lorazepam |
| **Opioids** n=13 | Codeine-Paracetamol (4) Tramadol (3) | Buprenorphine Morphine Oxycodone | Buprenorphine Morphine Oxycodone |  |
| **Other drugs** | **Alcohol** | **Cannabis** | **Stimulants** | **Hallucinogens** |
| **Antimicrobials** n=21 | Ciprofloxacin (2) Terbinafine (2) Amoxicillin Acyclovir Cephalexin Clindamycin Ivermectin Lymecycline Phenoxymethylpenicillin Rifaximin Sulfamethoxazole- trimethoprim | Terbinafine (2) | Terbinafine (2) Clindamycin Lymecycline Tetracycline | Terbinafine |
| **Cardiovascular** n=39 | Bisoprolol (3) Propranolol Hydrochlorothiazide (2) Furosemide Indapamide Spironolactone Amlodipine (4) Lercanidipine Candesartan (2) Losartan Atorvastatin Rosuvastatin Organic nitrate (2) Clopidogrel (2) Warfarin (2) Apixaban Ticagrelor | Enalapril (2) Acetylsalicylic acid Atorvastatin Bisoprolol Propranolol Organic nitrate Warfarin | Propranolol (2) Enoxaparin Simvastatin |  |
| **Non-opioid  analgesics** n=31 | Paracetamol (10) Ibuprofen (5) Acetylsalicylic acid (3) Etoricoxib (3) Naproxen (3) | Paracetamol (3) Ibuprofen (3) Acetylsalicylic acid |  |  |

Numbers in parentheses indicate repeated mentions of the same interaction pair.

**Supplementary table 2**. Most frequently mentioned drugs in bilateral drug-drug interaction questions (n = 76; some with multiple interaction pairs).

|  | **Antidepressants** | **Antiepileptics** | **Antipsychotics** | **Benzodiazepines and related drugs** | **Cardiovascular** | **Opioids** | **Alcohol / Nicotine withdrawal** | **Other  drugs** |
| --- | --- | --- | --- | --- | --- | --- | --- | --- |
| **ADHD-drugs** |  |  |  |  |  |  |  |  |
| Methylphenidate | Sertraline  Venlafaxine |  | Levomepromazine | Diazepam Clonazepam | Propranolol | Buprenorphine(+naloxone)  Tramadol | Disulfiram |  |
| **Antidepressants** |  |  |  |  |  |  |  |  |
| Bupropion | Amitriptyline (2)  Mirtazapine Doxepin | Pregabalin |  | Clonazepam | Bisoprolol | Buprenorphine (3)  Tramadol | Varenicline |  |
| Es/citalopram |  |  | Risperidone  Quetiapine | Diazepam (2)  Zolpidem | Propranolol | Buprenorphine |  |  |
| Fluoxetine |  |  |  |  |  | Codeine(+Paracetamol)  Tramadol |  | Tizanidine |
| Mirtazapine | Es/citalopram |  |  | Midazolam | Candesartan  Propranolol |  | Varenicline |  |
| Moclobemide | Daboxetine |  |  | Clonazepam | Valsartan | Buprenorphine |  |  |
| Venlafaxine |  |  |  |  |  | Buprenorphine  Tramadol | Disulfiram |  |
| **Antiepileptics** |  |  |  |  |  |  |  |  |
| Gabapentin |  |  |  |  |  |  |  | Orphenadrine |
| Pregabalin | Duloxetine |  | Quetiapine | Clonazepam  Diazepam | Propranolol  Warfarin | Codeine(+Paracetamol)  Tramadol |  | Hydroxyzine |
| Valproate |  |  |  |  |  | Buprenorphine(+naloxone) |  |  |
| **Antipsychotics** |  |  |  |  |  |  |  |  |
| Risperidone |  |  |  | Oxazepam |  |  |  |  |
| Quetiapine | Mianserin |  | Aripiprazole |  |  | Buprenorphine (3) | Disulfiram |  |
| **Benzodiazepines and related drugs** |  |  |  |  |  |  |  |  |
| Alprazolam | Citalopram (2) |  |  | Lorazepam | Bisoprolol  Lercanidipine |  |  | Levothyroxine  Omeprazole |
| Clonazepam |  |  |  |  |  |  | Disulfiram (2) |  |
| Diazepam |  | Gabapentin |  | Temazepam | Propranolol | Buprenorphine  Codeine(+Paracetamol) | Disulfiram |  |
| Oxazepam |  | Pregabalin | Quetiapine | Clonazepam |  |  | Disulfiram | Baclofen |
| Zopiclone |  |  |  |  |  |  | Disulfiram (2) | Carbimazole |
| **Opioids** |  |  |  |  |  |  |  | Hydroxyzine |
| Codeine |  |  |  |  |  |  |  | Tizanidine |
| Hydromorphone |  |  |  |  |  | Buprenorphine |  |  |
| Methadone |  |  |  |  |  | Buprenorphine |  |  |
| Oxycodone |  |  |  |  |  | Buprenorphine |  |  |
| Tramadol |  | Gabapentin |  |  |  | Buprenorphine (4)  Codeine(+Paracetamol) (3) | Naltrexone | Diclofenac  Meloxicam |

Numbers in parentheses indicate repeated mentions of the same interaction pair.
